# Multiple HIV-1 infections with evidence of recombination in heterosexual partnerships in a low risk Rural Clinical Cohort in Uganda

**DOI:** 10.1016/j.virol.2010.12.025

**Published:** 2011-03-01

**Authors:** Deogratius Ssemwanga, Frederick Lyagoba, Nicaise Ndembi, Billy N. Mayanja, Natasha Larke, Shuyi Wang, Joshua Baalwa, Carolyn Williamson, Heiner Grosskurth, Pontiano Kaleebu

**Affiliations:** a MRC/UVRI Uganda Research Unit on AIDS, P.O. Box 49 Entebbe, Uganda; b MRC Epidemiology Group, London School of Hygiene and Tropical Medicine, London, UK; c Department of Medicine and Microbiology, University of Alabama at Birmingham, Birmingham, AL, USA; d University of Cape Town, Faculty of Health Sciences, Cape Town, South Africa

**Keywords:** HIV-1, Multiple infections, Superinfection, Coinfection, Recombination

## Abstract

We report on the frequency of multiple infections, generation of recombinants and consequences on disease progression in 35 HIV-1 infected individuals from 7 monogamous and 6 polygamous partnerships within a Rural Clinical Cohort in Uganda. The *env*-C2V3, *gag*-p24 and *pol*-IN genes were sequenced. Single genome amplified half genome sequences were used to map recombination breakpoints. Three participants were dually infected with subtypes A and D, one case with subtype A and A/D recombinant and the fifth with 2 phylogenetically distinct A/D recombinants. Occurrence of A/D recombination was observed in two multiple infected individuals. Rate of late stage WHO events using Cox regression was 3 times greater amongst multiple infected compared to singly infected individuals (hazard ratio 3.35; 95% CI 1.09, 10.3; *p* = 0.049). We have shown that polygamous relationships involving subtype discordant partnerships was a major contributor of multiple infections with generation of inter subtype recombinants in our cohort.

## Introduction

The diversity of HIV-1 is greatest in Sub-Saharan Africa, with currently 9 subtypes, 48 Circulating Recombinant Forms (CRFs) and several Unique Recombinants Forms (URFs) identified (http://www.hiv.lanl.gov/content/sequence/HIV/CRFs/CRFs.html, March, 2010).

The high numbers of HIV-1 recombinants observed in these populations (Dowling et al., 2002; Gale et al., 2006; Harris et al., 2002; Herbinger et al., 2006; Hoelscher et al., 2002; Kiwanuka et al., 2008, 2009) might be an indication that infections with multiple subtypes are frequent. Such populations might provide opportunities to further understand how multiple infections and recombination occur and their consequences to HIV vaccine development, disease progression and scale up of anti-retroviral therapy.

Multiple infections (i.e. infection with more than one HIV-1 strain or subtype) could be as a result of co-infection (simultaneous transmission of two or more distinct viruses before a specific HIV-1 immune response is established) (Casado et al., 2007; Yerly et al., 2004) or superinfection (consecutive transmission of a second virus or other strains after seroconversion) (Altfeld et al., 2002; Blish et al., 2008; Fang et al., 2004; Jost et al., 2002; Jurriaans et al., 2008; Kozaczynska et al., 2007; Pernas et al., 2006; Piantadosi et al., 2007; Ramos et al., 2002; Smith et al., 2005a, 2005b, 2006; Streeck et al., 2008).

Over the past few years, there have been several reports of existence of individuals with multiple infections (Altfeld et al., 2002; Blish et al., 2008; Fang et al., 2004; Gottlieb et al., 2004; Herbinger et al., 2006; Hoelscher et al., 2002; Jost et al., 2002; Piantadosi et al., 2007, 2008; Sagar et al., 2003; Smith et al., 2005a; Streeck et al., 2008).

An earlier study in Mbeya region in Tanzania showed that the prevalence of multiple infections in the low and high risk sexual behaviour populations was 9% and 19% respectively (Herbinger et al., 2006). In Rakai, Uganda, a 5.8% prevalence of multiple infections was found in a low risk sexual behaviour cohort (Kiwanuka et al., 2009). Lower rates of multiple infection (3%) were also reported in a cohort of commercial sex workers in Burkina Faso (Manigart et al., 2004).

One consequence of HIV-1 multiple infections is recombination between the two or more different strains in the host. A few studies mostly based in one case each (Fang et al., 2004; Jobes et al., 2006; Kozaczynska et al., 2007; Munoz-Nieto et al., 2008; Pernas et al., 2006) have reported recombination in multiply infected individuals. This recombination could result in a fitter virus partly as a result of the evasion from host immune responses (Streeck et al., 2008). Multiple infections have also been shown to lead to faster disease progression (Gottlieb et al., 2004; Sagar et al., 2003). In our cohort which is a relatively low risk population, we observe a high percentage of unique recombinant viruses and it is of importance to understand how these arise.

Since 2002, there have been a number of reported cases of superinfection in different groups such as commercial sex workers, injection drug users and men who have sex with men (MSM) (Altfeld et al., 2002; Blish et al., 2008; Casado et al., 2007; Fang et al., 2004; Gottlieb et al., 2007; Gunthard et al., 2009; Jost et al., 2002; Koelsch et al., 2003; Manigart et al., 2004; McCutchan et al., 2005; Piantadosi et al., 2007, 2008; Rachinger et al., 2008; Ramos et al., 2002; Sidat et al., 2008; Smith et al., 2006; Smith et al., 2004; Smith et al., 2005a,b; Steain et al., 2004; Streeck et al., 2008; Yang et al., 2005; Yerly et al., 2004). Approximately half of the reported cases of superinfection were acquired within the first year after seroconversion. The incidence of superinfection was found to be 5% per year in a study in the USA amongst 78 ART-naïve individuals between 6 and 12 months after primary infection (Smith et al., 2004). On the other hand, a study in Kenya in 36 high risk sexual behaviour women who were followed up for 5 years after primary infection showed that 7 women were superinfected with a second virus (Piantadosi et al., 2007) and superinfection occurred throughout the course of the first infection.

Superinfection has been reported in individuals with cellular immune responses to the initial virus, indicating an inability or failure of immunity induced by the initial infection to confer protection against subsequent challenges (Altfeld et al., 2002; Blish et al., 2008; Piantadosi et al., 2007; Streeck et al., 2008). The role of neutralizing antibodies in superinfection is also unclear with one study indicating that low or non-existent broad neutralising antibody (NAb) responses to HIV-1 may allow superinfection (Smith et al., 2006), while another study (Blish et al., 2008) reported superinfection despite relatively robust neutralizing antibody responses.

We are currently undertaking a number of studies in various low and high risk cohorts in Uganda to characterize the host and viral factors associated with multiple infection and superinfection. In this study we report on the frequency of HIV multiple infections in a group of individuals in polygamous and monogamous sexual relationships. We report for the first time that polygamous relationships involving subtype discordant partnerships is a major contributor of multiple infections with a very efficient generation of inter subtype recombinants in our low risk rural cohort. The HIV disease progression amongst the multiple and singly infected participants is also assessed.

## Results

### Socio-demographic and clinical characteristics of the study population

The estimated seroconversion dates for the incident cases or known dates of enrollment for the prevalent cases and partner subtypes data was tabulated (Table 1). Data on socio-demographic, biological and clinical characteristics are given in Table 2. Age, sex, education and occupation, as measured at the first visit following seroconversion, were similar between the singly (*n* = 30) and multiple infected individuals (*n* = 5). The median viral load and CD4 count measured were also similar between these two groups at this visit (Table 2). An estimate of the seroconversion date and/or follow-up data was not available for four of these subjects and all subsequent analysis are restricted to the 31 subjects with an estimate of the seroconversion date.

### Determination of transmission linkage

To determine transmission linkages the phylogenetic relationships between *env-*C2V3 and *gag*-p24 were analysed in all the 7 monogamous and 6 polygamous partnerships (*n* = 35 participants). Transmission linkage was assumed if sequences reliably clustered together on phylogenetic trees (> 80% bootstrap support) with no other sequence being more closely related than the sequences were to each other. Cloning and sequencing was not done on all samples, but an average of 5–10 and 15–20 clones were sequenced in the *env*-C2V3 and *gag*-p24 genes per patient sample respectively. There was no end point dilution done prior to PCR.

**Fig. 1:**
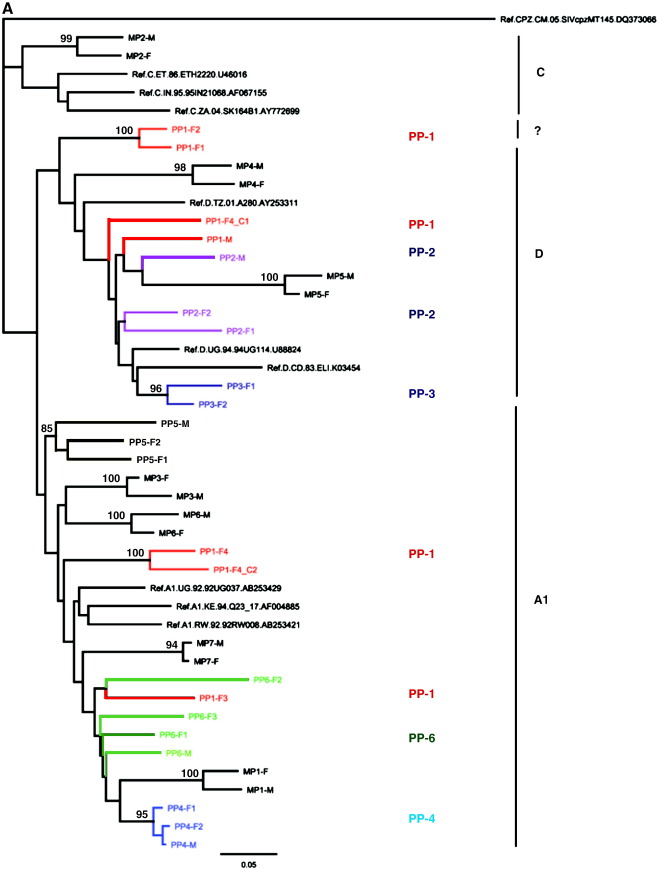

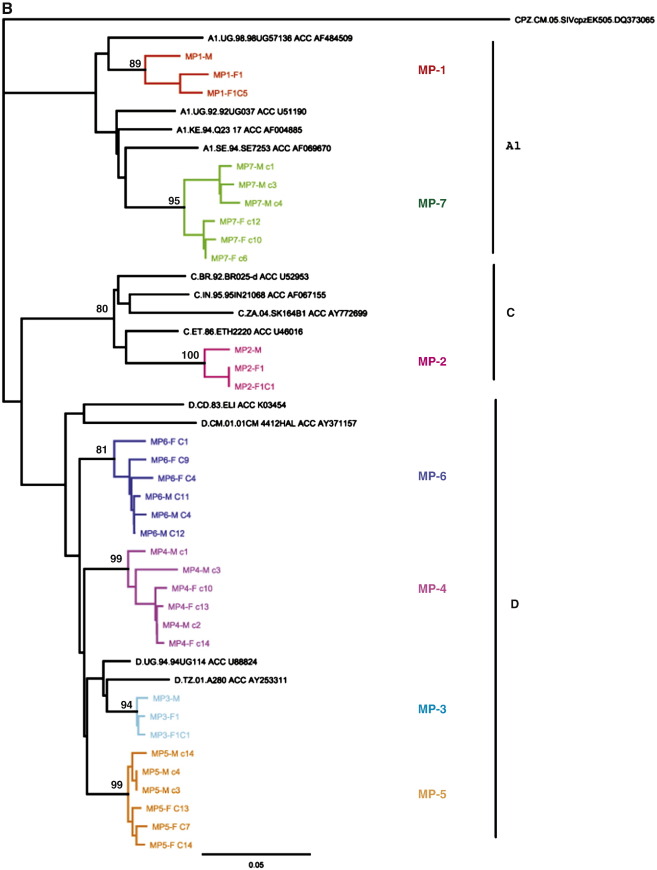

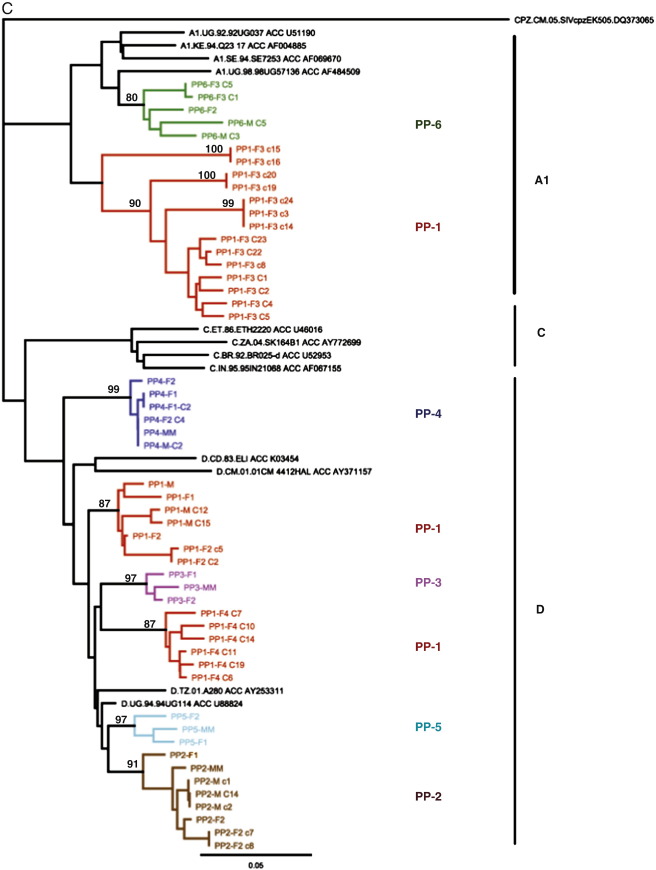

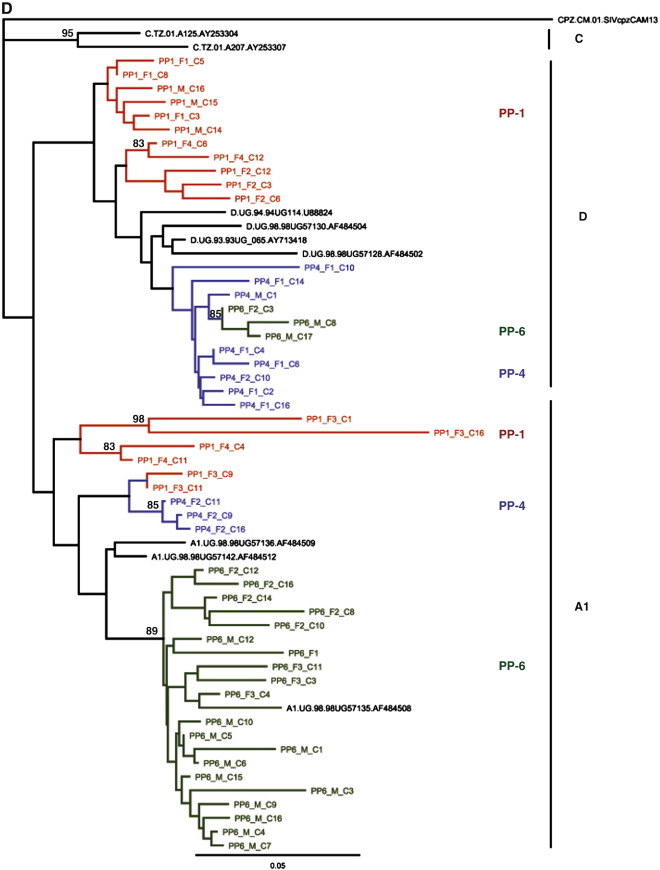
A. Phylogenetic analysis of the *env-*C2V3 sequences (HXB2 location 6829–7334) of Monogamous Partnerships (MP) 1–7 and Polygamous Partnerships (PP) 1–6. All PPs are coloured PP1 (red), PP2- (purple), PP3 (dark blue), PP4 (light blue), PP5 (black) and PP6 (green). Bootstrap values greater than 80% are shown. M—male partner and F—female partner. The phylogenetic plots do represent consensus sequences of the patients. B. Phylogenetic analysis of the *gag-*p24 sequences (HXB2 location 1123–1589) of Monogamous Partnerships (MP) 1–7. Bootstrap values greater than 80% are shown. M—male partner and F—female partner. The phylogenetic plots do represent consensus sequences of the patients. C. Phylogenetic analysis of the *gag-*p24 sequences (HXB2 location 1123–1589) of Polygamous Partnerships (PP) 1–6. Bootstrap values greater than 80% are shown. M—Male. D. Phylogenetic analysis of the *pol-*IN sequences in Polygamous Partnerships PP1, 4 and 6 (including clone and direct sequences HXB2 location 4470–4807). Bootstrap values greater than 80% are shown. M—male partner and F—female partner.

Transmissions were epidemiologically linked in all the 7 monogamous partnerships and 3 of the 6 polygamous partnerships analyzed (Table 1, Fig. 1A, B, C). Five of the seven monogamous partnerships were infected with pure subtypes based on *env*-C2V3/*gag*-p24 analysis including two infected with subtype A, two with subtype D and one with subtype C. Infection with A/D recombinant viruses was identified in two monogamous couples (Table 1, Fig. 1A, B). In the three linked polygamous partnerships, one was infected with subtype D and two had A/D recombinant viruses (Table 1, Fig. 1A, C).

Based on *env*-C2V3 sequencing, transmissions in the other 3 of the six polygamous partnerships were unlinked (Table 1, Fig. 1A). In the first discordant partnership, PP1 (one male and 4 females), *env*-C2V3 sequences from the husband (PP1-M) had a subtype D virus whereas the first and second wives (PP1-F1 and PP1-F2 respectively) were subtype discordant to their husband, both with a unique recombinant subtype D virus. The third wife (PP1-F3) was infected with a subtype A virus; and the fourth female (PP1-F4) in this household was found to be multiple infected with subtype A and D viruses. However *gag*-p24 sequences from the husband (PP1-M), first wife (PP1-F1) and second wife (PP1-F2) were all linked and were classified as subtype D infection; however the third wife (PP1-F3) was found to be multiple infected with two phylogenetically distinct strains of *gag*-p24 subtype A viruses; and the fourth female (PP1-F4) in this household was found to be infected with *gag*-p24 subtype D virus. (Table 1, Fig. 1C).

In the second discordant partnership, PP2, the husband (PP2-M) and the two wives (PP2-F1 and PP2-F2) were infected with unlinked subtype D viruses based on *env*-C2V3 sequencing (Table 1, Fig. 1A). However, based on *gag*-p24 sequencing, all partners in polygamous partnership 2 had linked subtype D viruses (Table 1, Fig. 1C).

In the third discordant partnership, PP6, the husband (PP6-M), first wife (PP6-F1), second wife (PP6-F2) and third wife (PP6-F3) all had unlinked *env*-C2V3 subtype A viruses (Table 1, Fig. 1A). Analysis of the *gag*-p24 sequences in polygamous partnership 6 showed that the husband (PP6-M), the second (PP6-F2) and third wife (PP6-F3) all had linked subtype A viruses. The *gag*-p24 sequence data for was unavailable for the first wife in polygamous partnership 6.

For quality control purposes, to be certain that patients from epidemiologically unlinked patients were not cross-contaminated, phylogenetic trees were constructed by including all direct and clonal sequences from the *env*-C2V3 (Sup. Fig. 1A) and *gag*-p24 genes (Sup. Fig. 1B).

### Transmission in unlinked partnerships: pol-IN analysis

To investigate if the unlinked partners had viruses transmitted to each other, the three polygamous partnerships that earlier showed either subtype discordance or recombinant viruses in the *env*-C2V3 and *gag*-p24 genes were analysed further in the *pol*-IN gene. The *pol*-IN gene that was analysed is a conservative 288 bp fragment that was ideal to identify multiple infections. Polygamous partnership 2 that had unlinked *env*-C2V3 and partnership 5 that had linked recombinant viruses were not analysed in the *pol*-IN due to insufficient sample volume. There was no end-point dilution done and between 15 and 20 clones were sequenced from each patient sample. Analysis of the three polygamous partnerships 1, 4 and 6 in the *pol-*IN by clonal sequencing confirmed one intrasubtype A and four intersubtype A/D multiple infections. In polygamous partnership 1, the husband (PP1-M) and the first wife (PP1-F1) were infected with a linked subtype D virus. The second wife (PP1-F2) had a discordant *pol-*IN subtype D virus whereas the third wife (PP1-F3) was found to be with two strains of *pol-*IN subtype A viruses. The fourth female (PP1-F4) in this household was also multiple infected with subtype A and D viruses (Table 1, Fig. 1D). Analysis of the *pol*-IN showed that there was transmission of viruses between the husband (PP1-M) and the first wife (PP1-F1) which were not detected in the other three partners who all had discordant viruses amongst themselves.

In polygamous partnership 4, the husband (PP4-M) and the two wives (PP4-F1 and PP4-F2) had linked *pol-*IN subtype D infections; in addition, a second *pol-*IN subtype A virus was detected in the second wife (PP4-F2) (Table 1, Fig. 1D) probably indicating that this female could have acquired this second virus from outside the partnership or that this second virus was not detectable in the other partners.

In polygamous partnership 6, the husband (PP6-M) and the three wives (PP6-F1, PP6-F2 and PP6-F3) were infected with linked *pol-*IN subtype A viruses. In addition to this, the husband (PP6-M) and the second wife (PP6-F2) were multiple infected with a second linked *pol-*IN subtype D virus (Table 1, Fig. 1D) also indicating that this second virus was not detected in the first female or that the husband and second wife acquired the second virus from outside of the partnership.

Analysis of the *pol*-IN amongst the subtype discordant partners shows that there is possible transmission of viruses amongst the partners and that there is a possibility of not detecting these viruses in some of the partners.

To be certain that patients from epidemiologically unlinked patients were not cross-contaminated, phylogenetic trees were constructed by including all direct and clonal sequences from the *pol*-IN gene for quality control (Sup. Fig. 1C).

### Single genome amplification assay (SGA)

**Fig. 2:**
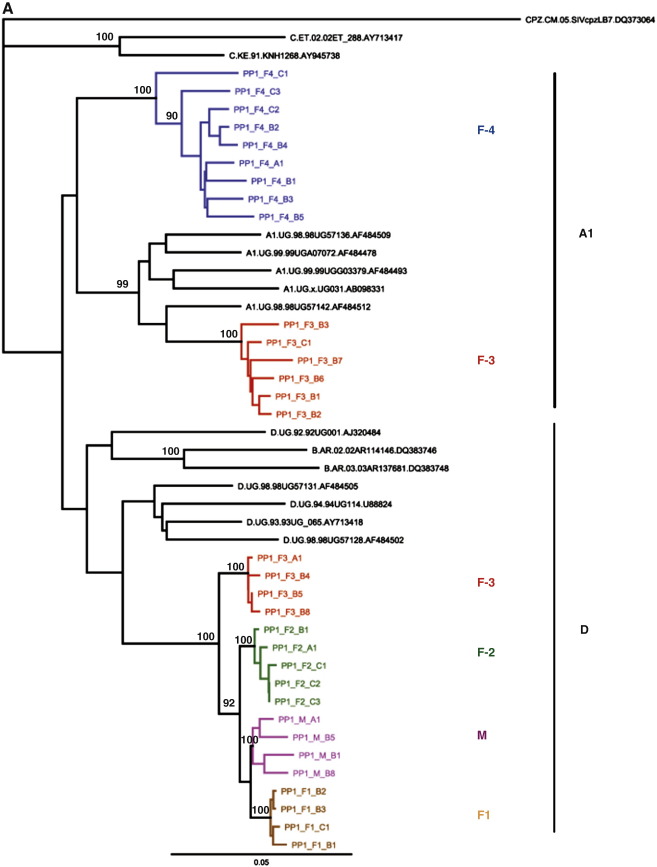

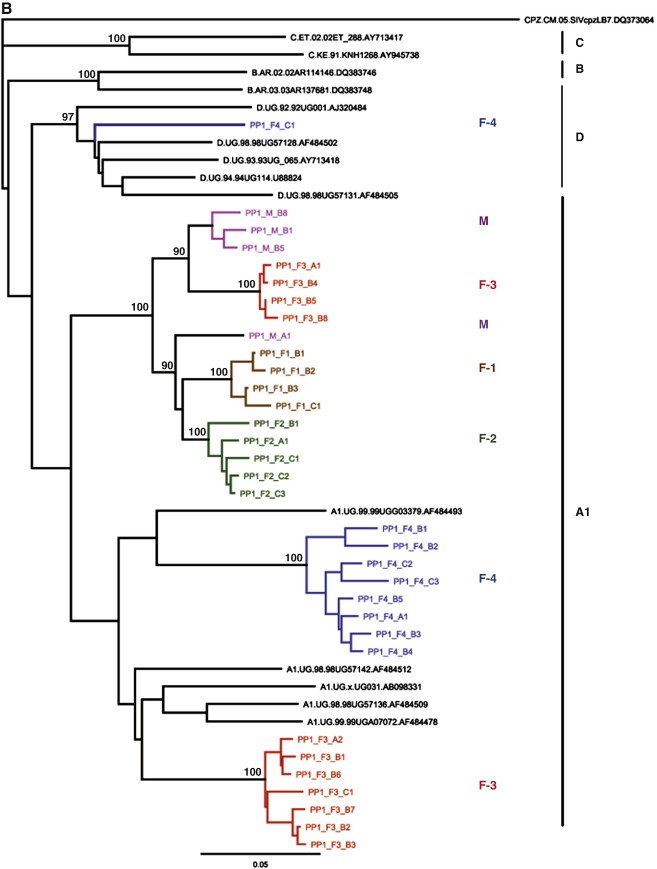

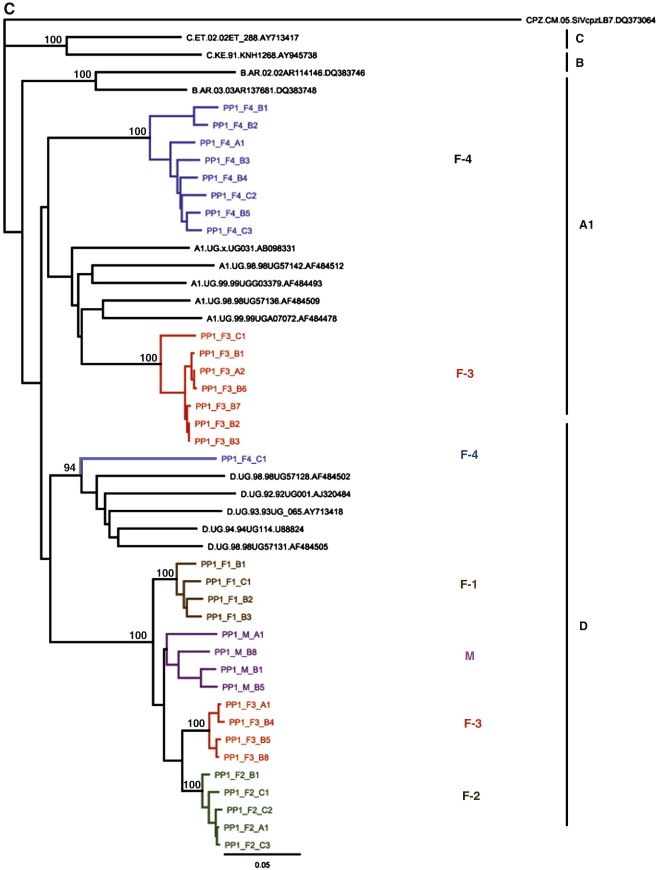

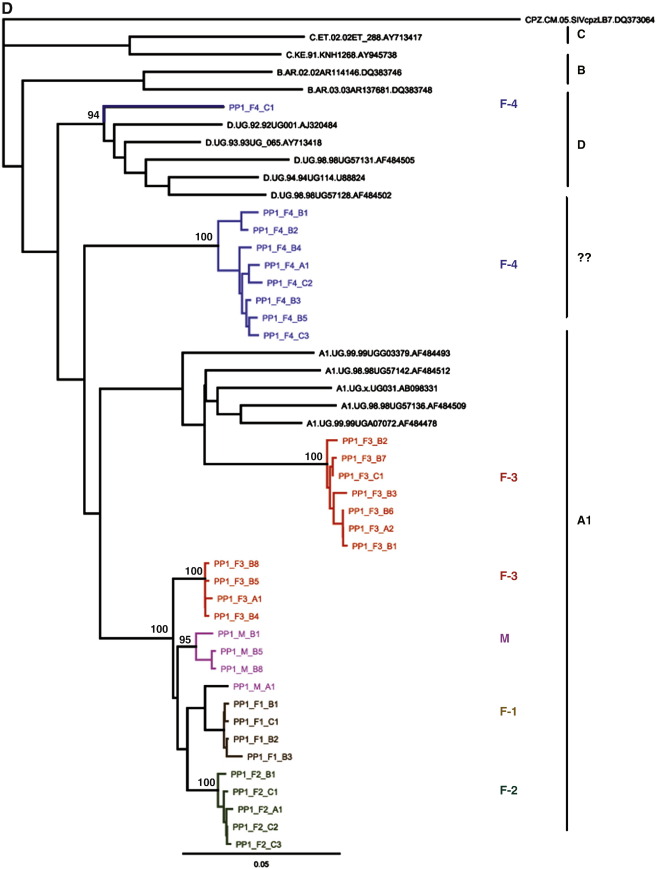

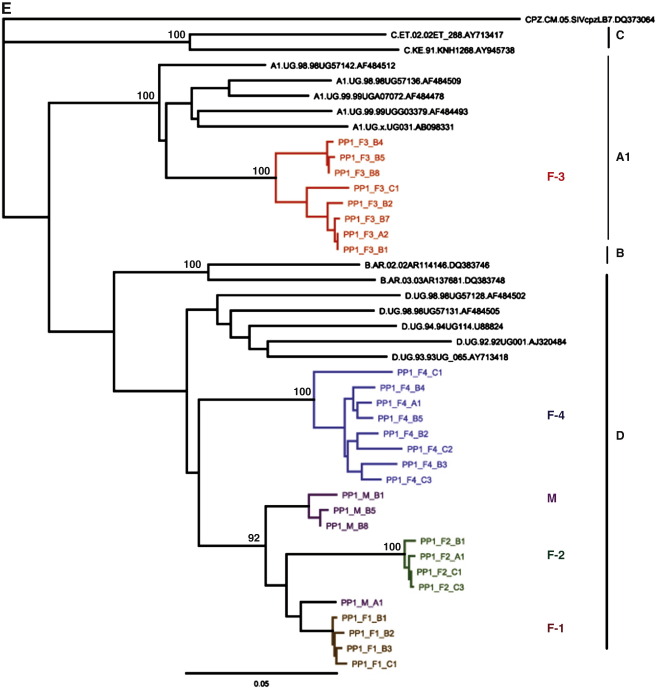
A. Phylogenetic analysis of the first SGA fragment (approx. 1000 bps) sequences in Polygamous Partnership 1. Bootstrap values greater than 90% are shown. M—male partner and F—female partner. B. Phylogenetic analysis of the second SGA fragment (approx. 1000 bps) sequences in Polygamous Partnership 1. Bootstrap values greater than 90% are shown. M-Male partner and F-Female partner. C. Phylogenetic analysis of the third SGA fragment (approx. 1000 bps) sequences in Polygamous Partnership 1. Bootstrap values greater than 90% are shown. M—male partner and F—female partner. D. Phylogenetic analysis of the fourth SGA fragment (approx. 1000 bps) sequences in Polygamous Partnership 1. Bootstrap values greater than 90% are shown. M—male partner and F—female partner. E. Phylogenetic analysis of the fifth SGA fragment (approx. 1000 bps) sequences in Polygamous Partnership 1. Bootstrap values greater than 90% are shown. M—male partner and F—female partner.

Two multiple infections earlier detected in *env*-C2V3, *gag*-p24 and *pol-*IN (intrasubtype A multiple infection PP1-F3 and intersubtype A/D multiple infection PP1-F4) were further confirmed following half genome SGA analysis. The SGA analysis performed on samples from polygamous partnership1 confirmed that the third wife (PP1-F3) was multiple infected with a pure subtype A virus and a recombinant A/D virus. (Table 1, Fig. 2A–E). The recombinant A/D virus had earlier not been detected in the *pol-*IN by cloning and sequencing. The fourth female (PP1-F4) was also confirmed to have two strains of A/D recombinant viruses with different recombination break points and it was shown that there was a possible recombination event between these two recombinants.

To analyse the recombinants based on a reference alignment of non-recombinant sequences, half genome sequences (HXB2 4778-9547) including the entire *env* gene were analysed in 5 fragments of a window size of approximately 1000 base pairs each. In the first fragment (Fig. 2), it was shown that the third wife (PP1-F3) was multiple infected with subtype A and D. Her subtype D virus was also linked to the husband's (PP1-M) and the other two wives' (PP-1-F1 and PP1-F2) viruses. The fourth female (PP1-F4) in this household had a unique recombinant subtype A virus in this fragment.

In the second fragment (Fig. 2B), the husband and three wives (PP1-F1, 2 and 3) had linked subtype A viruses. The third wife (PP1-F3) in addition had a second phylogenetically distinct subtype A virus. The two phylogenetically distinct strains of subtype A in PP1-F3 had been detected in *pol-*IN gene; thereby confirming it with SGA analysis. The fourth female (PP1-F4) in the household had two subtype A and D viruses. In the third fragment (Fig. 2C), the husband and three wives (PP1-F1, 2 and 3) had linked subtype D viruses. The third wife (PP1-F3) in addition had a subtype A virus. The fourth female (PP1-F4) was found with both subtype A and D viruses which was consistent with the observation in the *pol-*IN gene.

In the fourth fragment (Fig. 2D), the husband and three wives (PP1-F1, 2 and 3) had linked subtype A viruses. The third wife (PP1-F3) in addition had a second phylogenetically distinct subtype A virus. The fourth female (PP1-F4) in this household had two viruses; a unique recombinant subtype A and subtype D viruses. In the fifth fragment, the husband and two wives (PP1-F1 and 2) had linked subtype D viruses. The third wife (PP1-F3) had a subtype A virus and the fourth female (PP1-F4) had a discordant subtype D virus.

**Fig. 3:**
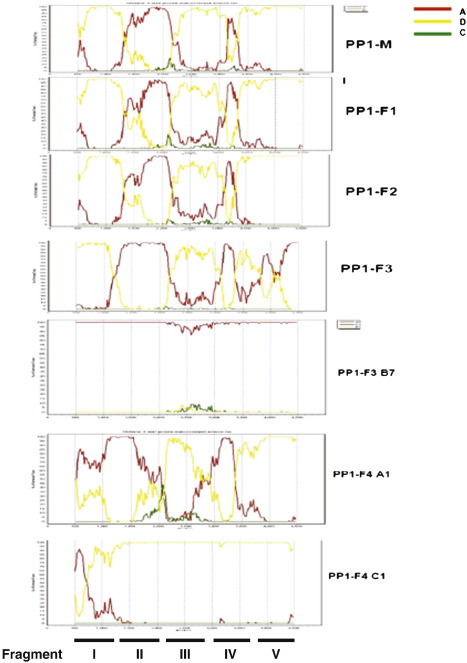
Bootscanning of half genome sequences (approx. 4800 bps) from the 5 partners in Polygamous Partnership 1 (PP1-M, PP1-F1, PP1-F2, PP1-F3 and PP1-F4). PP1-F3 and PP1-F4 each have 2 sequences and the other 3 partners each have one sequence. Query sequences are shown on the upper part of each partner's figure. Sequences to be compared with the query sequence are indicated on the right side of the figure. When comparisons were done, SimPlot generates a graph of percentage of permutated trees obtained using a sliding window of 200 nucleotides. The *y*-axis gives the percentage of permutated trees M—male partner and F—female partner. The fragments I–V analysed are shown at the bottom of the figure.

The bootscan analysis using pure reference sequences from subtypes A, D and C is shown in Fig. 3 which also shows the five fragments I–V that were phylogenetically analysed. In the fifth fragment, it was shown that in PP1-F3 only the parental pure subtype A virus was identified. The bootscan and phylogenetic trees show that there could have been recombination between the pure subtype A virus and the recombinant A/D virus in PP1-F3. There was also a possibility in the fourth female P1-F4 that there was recombination between the two viruses as the first and fifth fragments clustered together whereas the second, third and fourth fragments did not. To control for contamination, all sequences generated from the SGA were included in one phylogenetic tree (Sup. Fig. 1D).

### Adverse outcomes following seroconversion amongst singly and multiple infected individuals

The median follow-up was 8 years (IQR 4, 14) amongst singly infected individuals and 12 years amongst multiple infected individuals (IQR 7, 16). The median time to beginning ART or progressing to a low CD4 count (≤ 250) following seroconversion was similar amongst singly and multiple infected individuals: the median time to beginning ART was 11 vs. 13 years for multiple and singly infected individuals respectively (log rank *p* = 0.572); the median time to low CD4 count was 4 years vs. 6 years for multiple and singly infected individuals respectively (log rank *p* = 0.200). Conversely the median time to experiencing WHO disease events (either two stage 3 events or any single stage 4 event) (11 years for singly infected individuals) was significantly worse amongst multiple infected individuals (6 years) (log rank *p* = 0.026). Median time to death could not be calculated, and no difference was observed in mortality between singly and multiple infected individuals (log rank *p* = 0.713).

The rate of late stage WHO events (recurrent WHO stage 3 events or a single WHO stage 4 event) was approximately 3 times greater amongst multiple infected individuals compared to singly infected individuals (hazard ratio 3.35; 95% CI 1.09, 10.3; *p* = 0.049) (Table 3a). We chose the category of WHO stage 4 or repeat stage 3 events because this is used as criteria for commencing treatment in Uganda. The mortality rate, rate of progressing to a low CD4 count (≤ 250) and rate of commencing ART were also higher in the multiply infected individuals compared to singly infected individuals although there was no statistical evidence for these difference (Table 3a).

### Adverse outcomes following seroconversion amongst singly and multiple infected individuals restricted to only incident cases

The median follow-up was 7 years (IQR 4, 12) amongst singly infected individuals and 10 years amongst multiple infected individuals (IQR 6, 14). The median time to death, beginning ART or progressing to a low CD4 count (≤ 250) following seroconversion was similar amongst singly and multiple infected individuals: the median time to death was 14 years in singly infected individuals and 8 years in multiple infected individuals (log rank *p* = 0.445); the median time to beginning ART was 11 vs. 10 years for multiple and singly infected individuals respectively (log rank *p* = 0.805); the median time to low CD4 count was 4 years vs. 6 years for multiple and singly infected individuals respectively (log rank *p* = 0.390). Conversely the median time to experiencing WHO disease events (either two stage 3 events or any single stage 4 event) (10 years for singly infected individuals) was significantly worse amongst multiple infected individuals (5 years) (log rank *p* = 0.011) (Table 3b).

### Viral load and CD4 counts following seroconversion

**Fig. 4:**
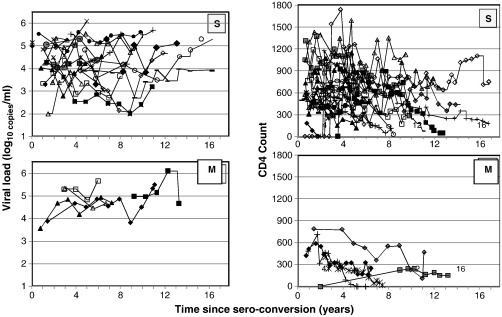
Viral load and CD4 count following HIV seroconversion. Only measurements taken prior to initiation of ART are depicted. Viral load (log_10_ copies/ ml) is plotted against time since seroconversion (years) in the left panels. No viral load measurements taken for five singly infected individuals. CD4 count is plotted against time since seroconversion (years) in the right hand panels. CD4 measurements were not available for one singly infected individual; for a further 3 singly infected individuals, CD4 measurements were not available prior to initiation of ART. S = singly infected individuals; M = multiple infected individuals.

We plotted the log_10_ viral load and CD4 count against time since seroconversion for the 27 singly infected and 5 multiple infected individuals (Fig. 4.). Only measurements prior to the initiation of ART were included. There was a trend showing that the viral load increased over time more rapidly amongst the multiple infected individuals and a decrease in CD4 count amongst multiple infected individuals appeared to occur at a more rapid pace compared to singly infected individuals. We also observed some two singly infected individuals that maintained their viral loads in the elite controller range of 100–1000 copies. We further observed that some individuals had viral loads that fluctuate, an observation noted over the years in other participants in our cohorts. We were however unable to investigate the host and viral factors that would explain this observation.

## Discussion

As part of on-going studies, to determine the frequency of multiple infections and super infections in our cohorts and relate this to host and viral factors we investigated the contribution of monogamous and polygamous partnerships. We have described the HIV transmission dynamics in couples in a rural Ugandan cohort and found that while monogamous partners had linked transmissions, three of the six polygamous partnerships, involving three men and nine women in total, were unlinked. We have identified multiple infections and in two individuals we find complex recombinant infections where there is evidence of a shared virus infection between the man and three other females in the partnership. We report the contribution of such partnerships to the generation of recombination viruses in this population.

In four of the seven monogamous partnerships, all with phylogenetically linked viruses, transmission was from the males to the females as the males had seroconverted before the females. However, in five of the polygamous partnerships, there was a possibility that transmissions were from one of the females who seroconverted before all the other partners. It is possible that more multiple infections in our study may have gone undetected due to techniques used in identification of multiple infections and viral dynamics during the course of infection. Indeed in our study, we identified more multiple infections when we combined both cloning and SGA techniques implying that to detect multiple infections, several genes must be analysed with the appropriate techniques.

All the subtype discordant viruses were observed in disclosed polygamous partnerships and this partly explained why all the multiple infections that were detected were from polygamous partnerships. We were however unable to determine whether these multiple infections were due to co-infection with two or more viruses simultaneously or due to super-infection since retrospective samples had earlier been exhausted.

The observed discordance amongst the polygamous partners may be due to polygamous partnerships formed after HIV infection, as in the case of partnership 1 where the third wife (PP1-F3) is discordant with a subtype A/A/A virus (*env*-C2V3*/gag*-p24*/pol*-IN) as opposed to the other two wives' and husband's subtype D/D/D viruses (*env*-C2V3*/gag*-p24*/pol*-IN). This observation was supported further by the third wife (PP1-F3) having a dual infection with a recombinant A/D virus and a pure subtype A virus. The subtype A virus was not detected in the other partners but the recombinant A/D was detected in the other three partners. This however does not rule out the possibility of HIV negative partners acquiring viruses from different sources outside the partnership. This may be a possible explanation for the observed discordance in one gene and concordance in another gene as was the case in PP2 and PP6. The absence of linkages in each individual may probably imply acquisition of other viruses outside the sexual network, however this is speculative. The *env*-C2V3 average genetic distance in PP2 and PP6 was 17% and 15% respectively.

The multiple infections amongst the polygamous partners may be an indication that the polygamous individuals have more partners than reported, further exposing them to super-infections. In this cohort over the years, partnerships have been formed between already HIV positive individuals (unpublished data) however no study has previously been undertaken to assess whether re-infections do occur and the prevalence of multiple infections amongst such individuals. Individuals in a known monogamous or polygamous sexual network have also been reported to move from one sexual partnership to another within the cohort (unpublished data). An in-depth analysis on these individuals to determine transmission dynamics has not been done. Another possible explanation for the observed discordance may be that partners acquire infections from outside the formal partnership (Trask et al., 2002). It is also however possible that we may have missed detecting some viruses in some partners that were found to be discordant due the methods used to detect multiple infections.

We have been able to identify five cases of multiple infections in our study, an observation that has been made in other studies (Gerhardt et al., 2005; Kozaczynska et al., 2007; Pernas et al., 2006; Takehisa et al., 1997, 1999; van der Kuyl et al., 2005). In our study, two individuals that were dually infected possibly resulted into recombination events. Our report further supports earlier studies that multiple infections are indeed frequent when there is exposure. Further still triple infection with two strains of subtype A and subtype C was reported in Tanzania (Gerhardt et al., 2005); in Cameroon, infection with a group O virus, a subtype D virus and subtype A/G recombinant virus was reported (Takehisa et al., 1997, 1999). In Spain, an individual was found to have three phylogenetically distinct strains of subtype B (Pernas et al., 2006) and in Netherlands an individual was found to harbour two strains of subtype B and CRF01_AE (Kozaczynska et al., 2007; van der Kuyl et al., 2005).

If the observed multiple infections were due to superinfection (which we were unable to determine) in the presence of an immune response this raises a big challenge to the development of a vaccine. There have been reports of superinfection in the presence of a cellular immune response (Altfeld et al., 2002; Jost et al., 2002; Streeck et al., 2008; Yang et al., 2005). The role of neutralizing antibodies in superinfection is also unclear with one study indicating that low or non-existent broad neutralising antibody (NAb) responses to HIV-1 may allow superinfection (Smith et al., 2006), while another study (Blish et al., 2008) reported superinfection despite relatively robust neutralizing antibody responses.

As previously shown by others (Altfeld et al., 2002; Gunthard et al., 2009; Hu et al., 2005; Jost et al., 2002; Ramos et al., 2002), our study has shown both intrasubtype and intersubtype multiple infections, which raises further concern of not only failure of cross subtype protection but even intrasubtype protection.

There are previous reports that have shown recombination of viruses amongst individuals with multiple infections (Fang et al., 2004; Jobes et al., 2006; Kozaczynska et al., 2007; Munoz-Nieto et al., 2008; Pernas et al., 2006). Our study further shows that there is a possibility of on-going recombination amongst individuals with multiple infections. This recombination due to multiple infections was shown to have possibly occurred.

Recombination following multiple infections and the outgrowth of the parental strains could also account for the failure to detect more multiple infections in our study (Peeters et al., 1999; Songok et al., 2004). If the super infecting strain is also transient with low levels of replication, then this will most likely be undetected (Yerly et al., 2004).

Multiple infections in some individuals was possibly detected in only one genome region, an observation that has been reported to be due to superinfection followed by recombination (Piantadosi et al., 2007). This study in Kenya amongst 36 high risk women showed seven cases of superinfection of which five cases had superinfection detected by differences in only one gene. Further longitudinal analysis of these five showed that superinfection was followed by recombination which resulted in detection of both the recombinant virus co-circulating with the first infecting virus. In our observation, there is a possibility that superinfection occurred that was followed by recombination which resulted in detection of multiple infections in only one gene like it was the case in the Kenyan study.

The clinical significance of multiple infections needs to be further investigated; apart from the previously reported effects on disease progression, multiple infections could complicate antiretroviral therapy if already infected drug naive individuals get superinfection with ART resistant viruses as earlier reported (Bezemer et al., 2008; Smith et al., 2005b). Some reports indicate that some of the observed sudden viral rebounds in patients on therapy is due to superinfection with a drug resistant virus rather than drug resistance emerging in the primary infecting virus (Jurriaans et al., 2008). Our study, though based on small numbers has shown that multiple infections were associated with faster disease progression as previously described in other studies (Gottlieb et al., 2004; Sagar et al., 2003). In our study, multiple infected individuals had significantly higher rates of late stage WHO events compared to the singly infected individuals. In addition, viral loads increased and CD4 counts reduced more rapidly amongst the multiple infected individuals compared to the singly infected individuals.

## Conclusion

We report for the first time that polygamous relationships involving subtype discordant partnerships is a major contributor of multiple infections with a very efficient generation of inter subtype recombinants in our low risk rural cohort. These multiple infections are probably due to super infection as a result of partnerships created between already infected individuals or infections outside these partnerships. We have further shown differences in disease progression between singly and multiple infected individuals and the reasons for these differences need to be further explored.

## Materials and methods

### Study setting and participants

In 1989 the then Medical Research Council Programme on AIDS in Uganda established a General Population Cohort (GPC) encompassing approximately 5000 adults drawn from a cluster of 15 villages in rural Southwest Uganda. This cohort has been described in detail elsewhere (Mulder et al., 1994). In 1990 a random selection of one-third of seropositive adults identified in the initial GPC serosurvey round were invited to enroll into the Rural Clinical Cohort (RCC), previously called Natural History Cohort, as prevalent HIV cases (Morgan et al., 1997). In 1999, 10 more villages were added to the GPC bringing the total number of enrolled adults to about 10,000. Subsequent annual serosurveys of the GPC have identified new HIV-seropositive participants, who have been recruited as incident cases, the majority of whom have estimated dates of seroconversion. Once enrolled into the RCC they attend the study clinic every three months for clinical history, examination and blood sampling. In the RCC, HIV infected participants are encouraged to bring their partner(s) for voluntary counseling and testing and possible enrollment.

### Participants' selection

Blood samples were drawn from 35 HIV-1 infected randomly selected participants, from 7 monogamous partnerships (MP) and 6 polygamous partnerships (PP) in the RCC. In the PP, 4 partnerships had a husband with 2 wives, 1 partnership had a husband with 3 wives and another partnership had a husband with 4 wives. The participants either had estimated seroconversion dates for the incident cases or known dates of enrollment for the prevalent cases. The seroconversion dates for the incident cases were estimated as the midpoint between the date of the last sero-negative test and the date of the first sero-positive test.

### RNA extraction and RT-Nested PCR

Viral RNA was extracted from cryo-preserved serum/plasma using QIAamp Viral RNA Mini Kit (Qiagen, Hilden, Germany). Reverse transcription and first round PCR was done using QIAGEN OneStep RT-PCR Kit (Qiagen, Hilden, Germany) to amplify the *env*-C2V3 and *gag*-p24 genes using universal primers. For the analysis of the *env*-C2V3, *gag*-p24 and *pol*-IN, end-point dilution was not performed prior to PCR, cloning (designated “c” on the phylogenetic tree) and sequencing.

### Envelope analysis

First round PCR primers ED31 forward (5′ CCT CAG CCA TTA CAC AGG CCT GTC CAA AG3′, at positions 6816–6844 of the HXB2 genome) and primer ED33 reverse (5′ TTA CAG TAG AAA AAT TCC CCT C 3′, at positions 7359–7380 of the HXB2 genome) (Delwart et al., 1995) and second round primers Bf forward (5′ TAA CAC AAG CCT GTC CAA AGG T 3′, at positions 6829–6847 of the HXB2 genome) and Br reverse (5′ AAT TTC TAG GTC CCC TCC TGA 3′, at positions 7317–7334 of the HXB2 genome) (unpublished) were used to amplify a 505-bp *env* gene product encompassing the *env-*C2V3 region.

The RT-PCR reaction contained; 10 μl 5× buffer, 2 μl dNTP mix, 2 μl Taq enzyme mix, 0.5 μl RNase inhibitor, 3 μl 10uM Primer ED31, 3 μl 10 μM Primer ED33, 4.5 μl water and 25 μl of RNA. The cycling conditions were 50 °C 30 min; 95 °C 15 min (95 °C 30 sec 54 °C 30 sec 72 °C 90 sec) ×40, 72 °C 10 min and 4 °C hold. The second round PCR reaction contained 10 μl 10X PCR buffer, 4 μl 10 mM dNTP mix, 10 μl 1.5 mM MgCl_2_, 2 μl of each 10 mM Primer Bf/Br, 0.6 μl Taq Polymerase, 61.4 μl dH_2_O and 10 μl cDNA. The cycling conditions were 1 cycle of 94 °C for 5 min, 35 cycles of 94 °C 10 sec, 50 °C 45 sec, 72 °C 1 min, then 1 cycle of 72 °C 10 min and a final hold at 4 °C.

### Gag analysis

The *gag* gene was amplified using 10 μM first round PCR primers H1G777 forward (5′ TCACCTAGAACTTTGAATGCATGGG 3′, at positions 777–801 of the HXB2 genome) and primer H1P202 reverse (5′ CTAATACTGTATCATCTGCTCCTGT 3′, at positions 1874–1898 of the HXB2 genome) and second round 10 μM primers H1Gag1584 forward (5′ AAAGATGGATAATCCTGGG 3′, at positions 1123–1141 of the HXB2 genome) and primer g17 reverse (5′ TCCACATTTCCAACAGCCCTTTTT 3′, at positions 1566–1589 of the HXB2 genome) to generate a 460 bp fragment. The RT-PCR and second round were described elsewhere (Heyndrickx et al., 2000).

The *env*-C2-V3 and *gag* PCR products were visualised on a 1.5% agarose gel to confirm positive PCR amplification and purified using the QIAquick PCR Purification Kit (Qiagen, Hilden, Germany).

### Sequencing and phylogenetic analysis

The purified PCR products were directly sequenced in the sense and antisense directions with primers Bf and Br for *env*-C2-V3 and H1Gag1584 and g17 for *gag*. The DNA sequencing was carried out by di-deoxy chain termination method by an in-house sequencing method using a Beckman capillary sequencer and an ABI capillary sequencer. The chromatogram files were read using the Sequencher 4.6 program (GeneCodes, USA), and manually refined in Se-Al (available at http://evolve.zoo.ox.a.uk/software). Subtype reference sequences of HIV-1 group M available from the Los Alamos Sequence database (http://www.hiv.lanl.gov/content/sequence/NEWALIGN/align.html, 2008) were used to automatically align the generated sequences using ClustalX. The MEGA version 4.0 software package (Tamura et al., 2007) was used to perform phylogenetic analysis and the pairwise evolutionary distances were estimated using the HKY85 model (Hasegawa et al., 1985). The phylogenetic trees were constructed by neighbor joining (Saitou and Nei, 1987) and the reliability of tree topologies was estimated by bootstrap analysis (1000 replicates) (Felsentein, 1985). Epidemiologically linked transmissions were defined as those with bootstrap values > 80%. Sequences from the same partnership were given similar colors for clarity purposes on the phylogenetic trees. Our Laboratory is enrolled in two proficiency schemes such as Virology Quality Assurance and Quality Control for Molecular Diagnostics for genotyping.

### RT-PCR, clonal sequencing and phylogenetic analysis

Polygamous partners that had subtype discordant and recombinant viruses were analyzed further in the *pol-*IN gene. Viral RNA was extracted as earlier described and the *pol-*IN amplified using first round PCR primers Unipol5 (5′ TGGGTACCAGCACACAAAGGAATAGGAGGAAA-3′, at positions 3434–3765 of HIV-1LAI) and Unipol6 (5′ CCACAGCTGATCTCTGGCCTTCTCTGTAATAGACC 3′, at position 4483–4516 of HIV-1LAI) and second round primers Unipol1 (5′ AGTGGATTCATAGAAAGCAGAAGT 3′, at positions 4052–4074 of HIV-1LAI) and Unipol2 (5′ CCCCTATTCCTTCCCCTTCTTTTAAAA 3′, at positions 4363–4388 of HIV-1LAI) to generate a 288 nucleotide sequence fragment (Takehisa et al., 1998). The RT-PCR and second round reaction mixtures and thermocycle conditions were the same as the *env* and *gag* described above. The PCR products were cleaned as earlier described and cloned into pGEM-T Easy Vector Systems (Promega Corp., USA) and plasmids were transformed into High Efficiency Competent cells according to manufacturer's instructions. The transformants were cultured over-night on LB/Ampicillin/IPTG/X-Gal blue-white screening plates. Between 10 and 20 positive clones were selected for each sample from several PCR products and sequenced using Unipol1 and Unipol2 primer sets. For the purpose of clarity multiple clones were included for individuals with evidence of intra/inter-subtype multiple infections.

### Viral load measurements

HIV-1 RNA was tested using the Bayer VERSANT RNA 3.0 assay (lower limit of detection 50 copies/ml) for baseline samples, and the Roche Amplicor MONITOR 1.5 for other samples (lower limit of detection 400 copies/ml). The later assay replaced the former following successful demonstration of the correlation between the two assays.

### Single genome amplification assay (SGA)

Participants that were found to have multiple infections were analyzed further by SGA as described (Salazar-Gonzalez et al., 2008) to confirm multiple infections and recombination. In brief, cDNA was endpoint diluted in 96-well plates such that fewer than 29 PCRs yielded an amplification product. According to a Poisson distribution, the cDNA dilution that yields PCR products in no more than 30% of wells contains one amplifiable cDNA template per positive PCR more than 80% of the time. At least 10 half genome (positions 4768–9601 of the HXB2 genome) sequences were generated from each sample. Viral recombination analysis and location of breakpoints was done by bootscanning in Simplot software (Lole et al., 1999). A sliding window size of 1000 bp was used to analyse the 5000 bp half genome sequences. Recombination analysis was done to determine if partners had viruses with similar breakpoints or if there was recombination in the multiple infected individuals.

### Statistical analyses

Analysis was restricted to those individuals, for whom clinical data was available at more than one clinic visit. Socio-demographic, biological and clinical outcomes were compared between singly and multiple infected individuals using Fishers exact test or a Wilcoxon rank sum test. Median times to experiencing an adverse event (survival time) were estimates using Kaplan –Meier methods and a log-rank test was used to compare survival between groups. Cox regression was used to compare the rate of disease outcomes in the singly and multiple infected individuals; due to small numbers only univariate analyses were performed.

### Ethical considerations

The Uganda Virus Research Institute Science Ethics Committee as well as the National Council of Science and Technology approved the study. All participants provided written informed consent for participation.

From January 2004, antiretroviral therapy has been made available to all eligible HIV-infected patients according to the Uganda Ministry of Health National ART guidelines, (National Antiretroviral Treatment and Care Guidelines for Adults and Children. 1st Edition, 2003. Ministry of Health, Republic of Uganda).

Participants in the GPC and RCC are strongly encouraged to undergo voluntary HIV counseling and testing, and to disclose their HIV status to their partners.

## Accession numbers

The sequences have been deposited into Genbank under the accession numbers HM027653–HM027878HM027653HM027654HM027655HM027656HM027657HM027658HM027659HM027660HM027661HM027662HM027663HM027664HM027665HM027666HM027667HM027668HM027669HM027670HM027671HM027672HM027673HM027674HM027675HM027676HM027677HM027678HM027679HM027680HM027681HM027682HM027683HM027684HM027685HM027686HM027687HM027688HM027689HM027690HM027691HM027692HM027693HM027694HM027695HM027696HM027697HM027698HM027699HM027700HM027701HM027702HM027703HM027704HM027705HM027706HM027707HM027708HM027709HM027710HM027711HM027712HM027713HM027714HM027715HM027716HM027717HM027718HM027719HM027720HM027721HM027722HM027723HM027724HM027725HM027726HM027727HM027728HM027729HM027730HM027731HM027732HM027733HM027734HM027735HM027736HM027737HM027738HM027739HM027740HM027741HM027742HM027743HM027744HM027745HM027746HM027747HM027748HM027749HM027750HM027751HM027752HM027753HM027754HM027755HM027756HM027757HM027758HM027759HM027760HM027761HM027762HM027763HM027764HM027765HM027766HM027767HM027768HM027769HM027770HM027771HM027772HM027773HM027774HM027775HM027776HM027777HM027778HM027779HM027780HM027781HM027782HM027783HM027784HM027785HM027786HM027787HM027788HM027789HM027790HM027791HM027792HM027793HM027794HM027795HM027796HM027797HM027798HM027799HM027800HM027801HM027802HM027803HM027804HM027805HM027806HM027807HM027808HM027809HM027810HM027811HM027812HM027813HM027814HM027815HM027816HM027817HM027818HM027819HM027820HM027821HM027822HM027823HM027824HM027825HM027826HM027827HM027828HM027829HM027830HM027831HM027832HM027833HM027834HM027835HM027836HM027837HM027838HM027839HM027840HM027841HM027842HM027843HM027844HM027845HM027846HM027847HM027848HM027849HM027850HM027851HM027852HM027853HM027854HM027855HM027856HM027857HM027858HM027859HM027860HM027861HM027862HM027863HM027864HM027865HM027866HM027867HM027868HM027869HM027870HM027871HM027872HM027873HM027874HM027875HM027876HM027877HM027878.

The following are the supplementary materials related to this article.

Supp. Fig. 1A Phylogenetic analysis of all direct and cloned *env-*C2V3 sequences (HXB2 location 6829–7334) of Monogamous Partnerships (MP) 1–7 and Polygamous Partnerships (PP) 1–6. Bootstrap values greater than 80% are shown. M-Male partner and F-Female partner.

Supp. Fig. 1B Phylogenetic analysis of all direct and cloned *gag-*p24 sequences (HXB2 location 1123–1589) of Monogamous Partnerships (MP) 1–7 and Polygamous Partnerships (PP) 1–6. Bootstrap values greater than 80% are shown. M-Male partner and F-Female partner.

Supp. Fig. 1C Phylogenetic analysis of *pol-*IN sequences in Polygamous Partnerships PP1, 4 and 6 (including all clone and direct sequences HXB2 location 4470–4807). Bootstrap values greater than 80% are shown. M-Male partner and F-Female partner.

Supp. Fig. 1D Phylogenetic analysis of all env-SGA sequences in Polygamous Partnership ,4 and 6. Bootstrap values greater than 90% are shown. M-Male partner and F-Female partner.

Supp. Table 3a Rate of disease outcomes in multiple and singly infected individuals using any single WHO stage 3 event or any single WHO stage 4 event.

Supp. Table 3b Rate of disease outcomes in incident multiple and singly infected individuals any single WHO stage 3 event or any single WHO stage 4 event.

## Figures and Tables

**Table 1:** HIV-1 subtype distribution among partners.

Partner HIV-1 subtypes									
	Sample ID	SC/RP date	Sample date	Linked	Sequencing subtype				DI
*Monogamous Partnerships (MP)*									
Partnership1					***env***	***gag***	***pol***	***SGA***	
1	MP1-M	Feb 26, 03	Oct 18, 04	linked	**A**	**A**			
2	MP1-F	Jul 13, 04	Jul 12, 04	**A**	**A**			
Partnership2									
3	MP2-M	Feb 9, 93	Mar 2, 00	linked	**C**	**C**			
4	MP2-F	Jan 23, 02	Mar 13, 02	**C**	**C**			
Partnership3									
5	MP3-M	Oct 7, 02	Jun 22, 04	linked	**A**	**D**			
6	MP3-F	Nov 9, 99	Feb 22, 01	**A**	**D**			
Partnership4									
7	MP4-M	P NA	May 10, 05	linked	**D**	**D**			
8	MP4-F	Nov 7, 06	Oct 23, 08	**D**	**D**			
Partnership5									
9	MP5-M	Aug 23, 06	Feb 18, 09	linked	**D**	**D**			
10	MP5-F	P NA	Dec 11, 02	**D**	**D**			
Partnership6									
11	MP6-M	P NA	Jun 6, 07	linked	**A**	**D**			
12	MP6-F	Dec 2, 06	Nov 20, 08	**A**	**D**			
Partnership7									
13	MP7-M	Jan 6, 07	Dec 10, 08	linked	**A**	**A**			
14	MP7-F	P NA	May 4, 98	**A**	**A**			
								
*Polygamous Partnerships (PP)*									
Partnership1									
15	PP1-M	Mar 30, 97	Feb 7, 05	unlinked	**D**	**D**	**D**	**A/D**	
16	PP1-F1	Dec 3, 02	Jun 20, 05	linked	**D**	**D**	**D**	**A/D**	
17	PP1-F2	Mar 21, 01	Apr 9, 03	linked	**D**	**D**	**D**	**A/D**	
18	PP1-F3	Oct 23, 96	Apr 9, 03	unlinked	**A**	A+A*	A+A*	**A, A/D**	**DI**
19	PP1-F4	Jul 17, 93	Sep 15, 00	unlinked	**A+D**	**D**	**A+D**	**A/D, A/D**	**DI**
Partnership2									
20	PP2-M	P 01-Oct-91	Oct 8, 08	unlinked	**D**	**D**			
21	PP2-F1	P 20-Dec-90	Sep 24, 02	unlinked	**D**	**D**			
22	PP2-F2	Feb 22, 99	Aug 2, 00	unlinked	**D**	**D**			
Partnership3									
23	PP3-M	Mar 17, 96	Oct 17, 99	linked	**n/d**	**D**			
24	PP3-F1	Sep 8, 95	Jan 27, 00	**D**	**D**			
25	PP3-F2	Sep 9, 95	Jan 27, 00	**D**	**D**			
Partnership4									
26	PP4-M	Jan 14, 97	Nov 24, 99	linked	**A**	**D**	**D**	**D**	
27	PP4-F1	Feb 4, 99	Jul 6, 00	**A**	**D**	**D**	**D**	
28	PP4-F2	Mar 26, 96	Nov 17, 99	**A**	**D**	**A+D**	**D**	**DI**
Partnership5									
29	PP5-M	Nov 19, 91	Dec 6, 04	linked	**A**	**D**			
30	PP5-F1	Dec 24, 00	Aug 30, 01	**A**	**D**			
31	PP5-F2	Mar 3, 92	Oct 5, 99	**A**	**D**			
Partnership6									
32	PP6-M	P 8-Nov-90	Jul 13, 00	unlinked	**A**	**A**	**A+D**		**DI**
33	PP6-F1	P 6-Apr-04	Feb 7, 01	unlinked	**A**	**n/d**	**A**		
34	PP6-F2	Mar 16, 95	Aug 2, 00	unlinked	**A**	**A**	**A+D**	**A**	**DI**
35	PP6-F3	P 17-Jul-91	May 9, 07	unlinked	**A**	**A**	**A**	**A**	

MP—monogamous partnership.

M—male.

F—female.

PP—polygamous partnership.

n/d—not done.

DI—dual infection.

P—prevalent at enrollment.

SC Date—estimated seroconversion date (mid-point between last HIV sero-negative test and first HIV sero-positive date).

RP date—recruitment date for prevalent cases.

NA—not available.

SGA—single genome amplification assay.

Sample date—collection date of sample used for subtyping.

Transmission linkage based on env-C2V3.

**Table 2:** Socio-demographic characteristics, viral load and CD4 count at first HIV seropositive test.

Characteristic	Single infected	Multiple infected	*p* Value
	(*n* = 30)	(*n* = 5)	
*n* (%)	*n* (%)
Age^a^			
< 25 years	8 (36%)	2 (40%)	1.00
25 years or more	14 (64%)	3 (60%)	
Median age^a^ [IQR]	27 (23, 34)	25 (23, 43)	0.901
Sex			
Male	12 (40%)	1 (20%)	0.630
Female	18 (60%)	4 (80%)	
Education^b^			
No schooling	3 (13%)	0 (0%)	
Primary or Secondary education	21 (87%)	3 (75%)	
Higher education	0 (0%)	1 (25%)	0.075
Main occupation^b^			
Farmer	13 (54%)	2 (50%)	
Other	11 (46%)	2 (50%)	1.00
Infecting clade^c^ (*env*-C2V3/*gag*-p24)			
A	6 (20%)	n/a	n/a
C	2 (7%)	n/a	
D	10 (33%)	n/a	
A/D recombinant	12 (40%)	n/a	
A/ D multiple infection	n/a	5 (100%)	
Median viral load log_10_ copies/ ml, (95% CI)a, d	4.25 (3.76, 5.02)	4.98 (3.73, 5.52)	0.454
Median CD4 count [IQR]^e^	512 (145, 770)	425 (202, 636)	0.726

a Data missing for 8 singly infected individuals.

b Data missing for 6 singly infected individuals and 1 multiple infected individual.

c Data available for 30 singly infected individuals and 5 multiple infected individuals.

d First viral load measurement after seroconversion. This measurement was taken between 0 and 69 months after seroconversion for singly infected patients and between 9 and 110 months after seroconversion in multiple infected individuals.

e Data missing for 4 singly infected patients.
