# Supplementary figures and images for: Multiple HIV-1 infections with evidence of recombination in heterosexual partnerships in a low risk Rural Clinical Cohort in Uganda
Source: Virology. 2011 Mar 1;411(1):113–31. doi: 10.1016/j.virol.2010.12.025 (PMC3041926; doi:10.1016/j.virol.2010.12.025)

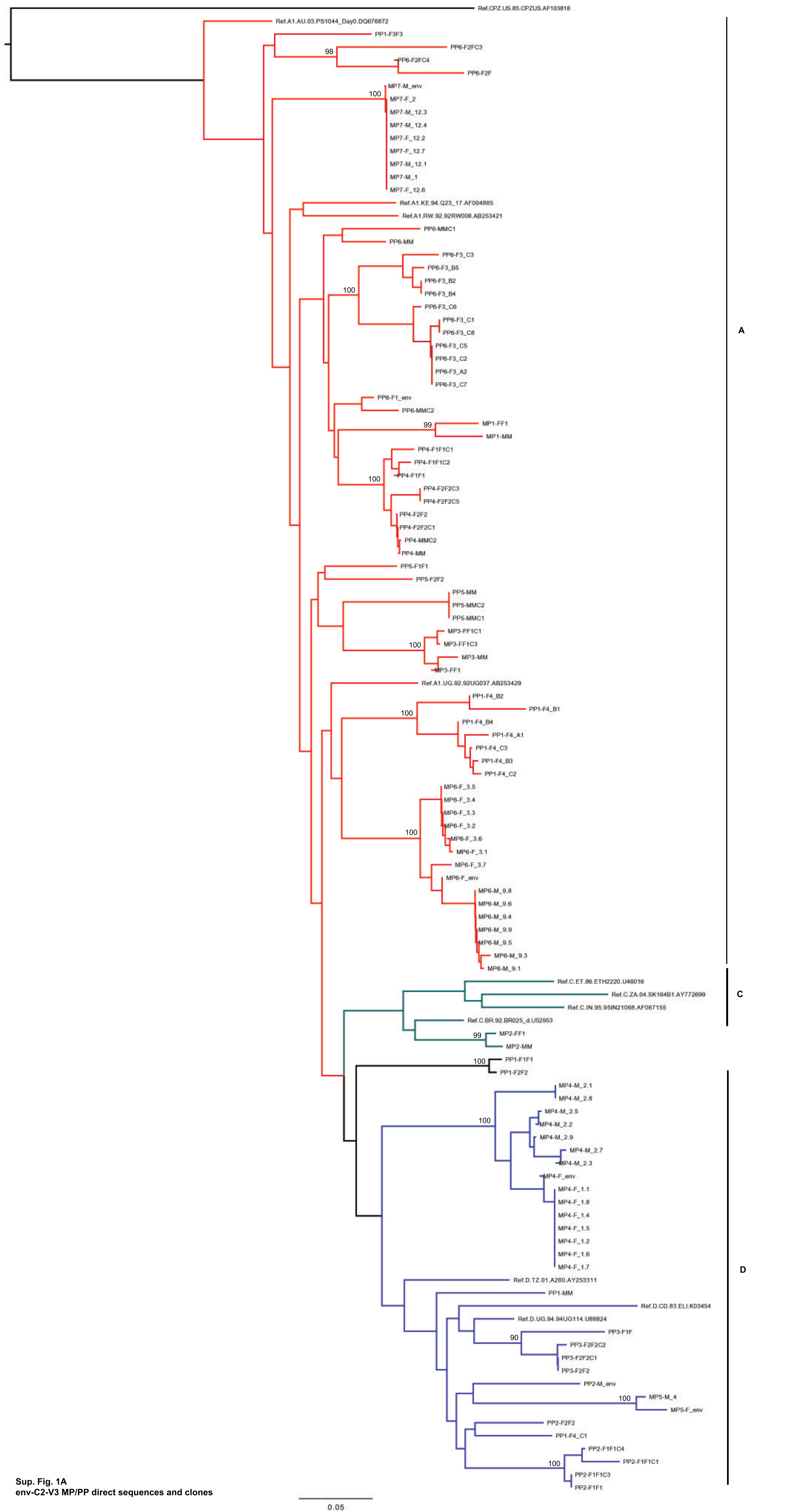

Supplement: Supp. Fig. 1A — Phylogenetic analysis of all direct and cloned env-C2V3 sequences (HXB2 location 6829–7334) of Monogamous Partnerships (MP) 1–7 and Polygamous Partnerships (PP) 1–6. Bootstrap values greater than 80% are shown. M-Male partner and F-Female partner. [file mmc1.pdf]

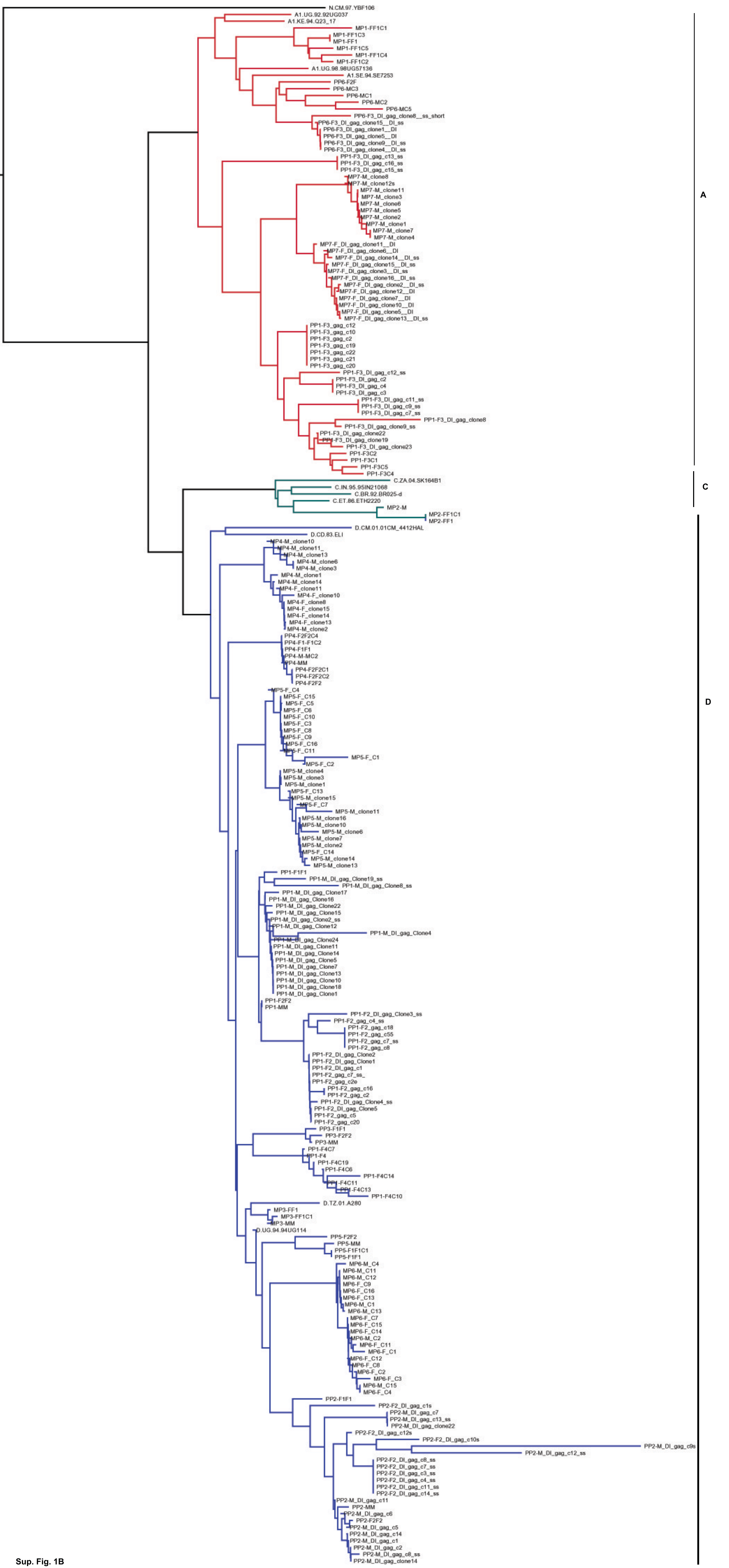

Supplement: Supp. Fig. 1B — Phylogenetic analysis of all direct and cloned gag-p24 sequences (HXB2 location 1123–1589) of Monogamous Partnerships (MP) 1–7 and Polygamous Partnerships (PP) 1–6. Bootstrap values greater than 80% are shown. M-Male partner and F-Female partner. [file mmc2.pdf]

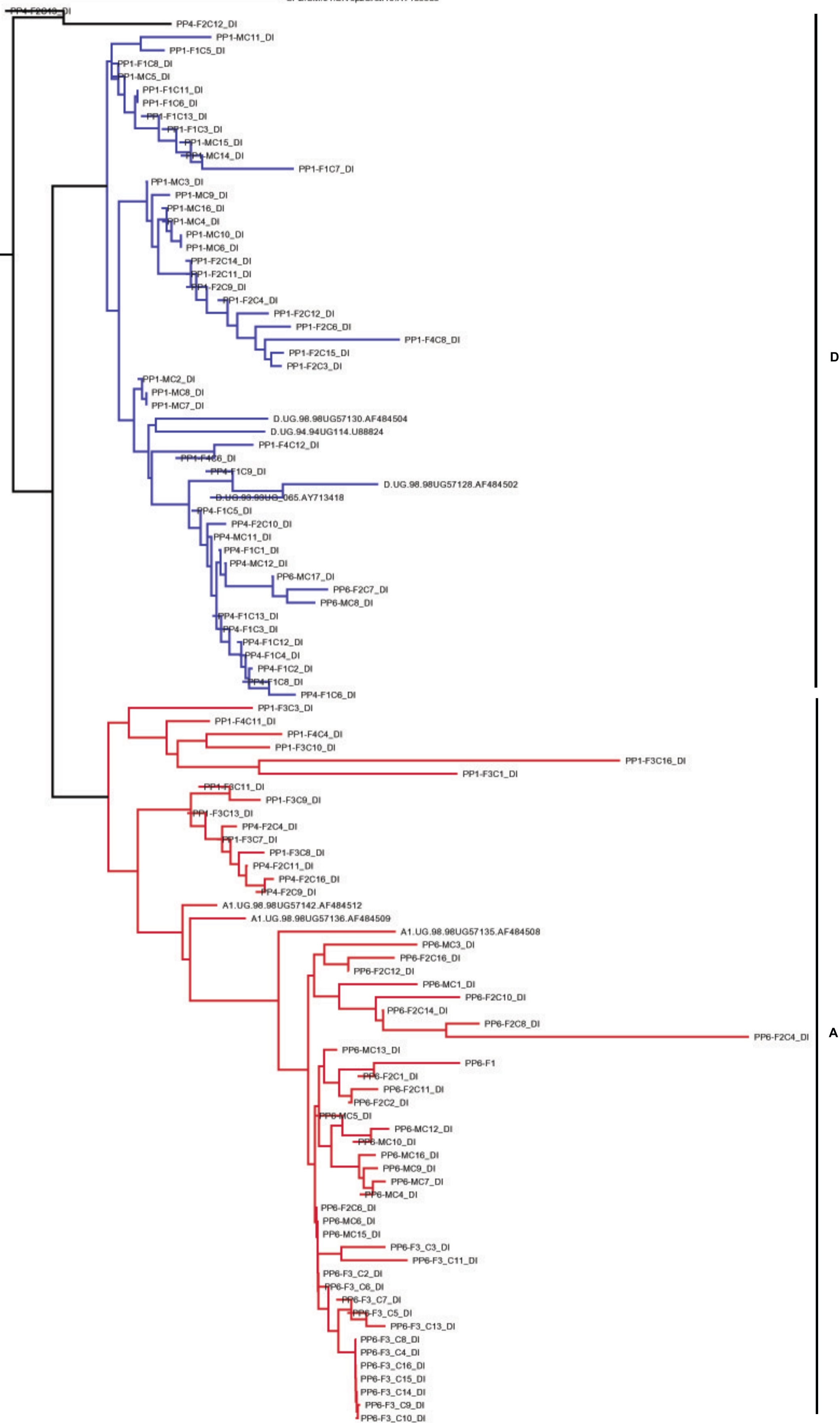

Sup. Fig. 1C  
pol-IN clones and direct sequences

0.03

Supplement: Supp. Fig. 1C — Phylogenetic analysis of pol-IN sequences in Polygamous Partnerships PP1, 4 and 6 (including all clone and direct sequences HXB2 location 4470–4807). Bootstrap values greater than 80% are shown. M-Male partner and F-Female partner. [file mmc3.pdf]

D

A

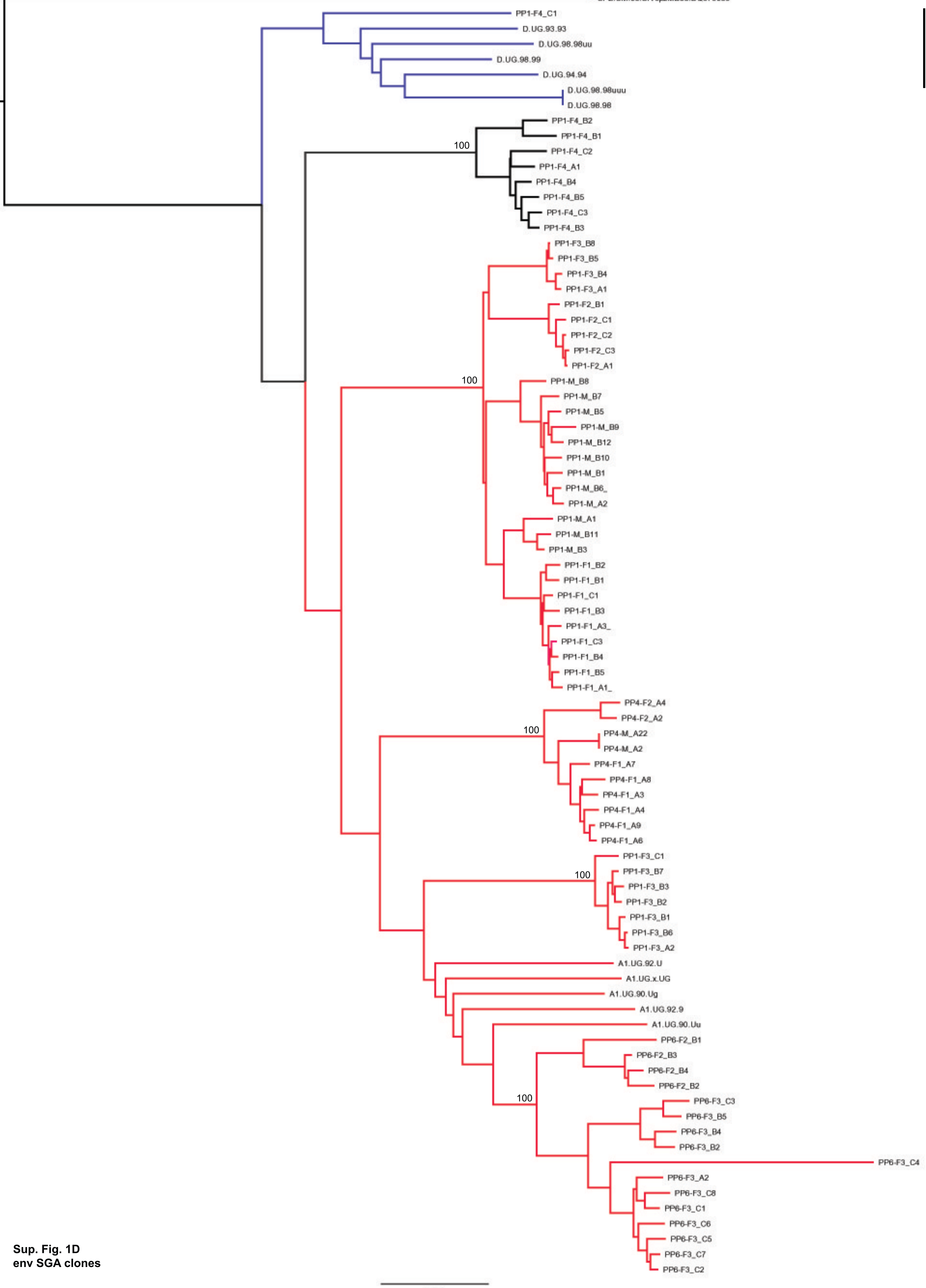

Sup. Fig. 1D  
env SGA clones

0.03

Supplement: Supp. Fig. 1D — Phylogenetic analysis of all env-SGA sequences in Polygamous Partnership ,4 and 6. Bootstrap values greater than 90% are shown. M-Male partner and F-Female partner. [file mmc4.pdf]
