# Supplementary material for: Multiple HIV-1 infections with evidence of recombination in heterosexual partnerships in a low risk Rural Clinical Cohort in Uganda
Source: Virology. 2011 Mar 1;411(1):113–31. doi: 10.1016/j.virol.2010.12.025 (PMC3041926; doi:10.1016/j.virol.2010.12.025)
Supplement: Supp. Table 3a — Rate of disease outcomes in multiple and singly infected individuals using any single WHO stage 3 event or any single WHO stage 4 event. [file mmc5.doc]

**Table 3a. Rate of disease outcomes in multiple and singly infected individuals**

|  | Singly infected  (N = 26) | | Multiple infected  (N = 5) | | Crude Hazard Ratio  (95% CI) | P value |
| --- | --- | --- | --- | --- | --- | --- |
|  | n (%) | Rate per  100y (95% CI) | n (%) | Rate per  100y (95% CI) |
| Mortality | 6 (23%) | 2.6 (1.2, 5.7) | 2 (40%) | 3.4 (0.9, 13.8) | 1.35 (0.27, 6.72) | 0.721 |
| CD4≤250^a^ | 13 (54%) | 10.1 (5.9, 17.5) | 5 (100%) | 20.8 (8.6, 49.9) | 1.95 (0.69, 5.52) | 0.233 |
| Received ART^b^ | 10 (42%) | 5.0 (2.7, 9.3) | 3 (60%) | 6.7 (2.1, 20.6) | 1.47 (0.39, 5.55) | 0.586 |
| WHO disease events^c, d^ | 13 (50%) | 15.7 (8.4-29.2) | 5 (100%) | 20.0 (8.3- 48.1) | 1.51 (0.48-4.71) | 0.48 |

^a^ Two singly infected individuals excluded from analysis as CD4 count≤250 at time of first HIV test

^b^ Two singly infected individuals excluded from analysis as started ART at time of first HIV test

^c^ One singly infected individuals excluded from analysis as experienced WHO stage events at time of first HIV test

^d^ Any single WHO stage 3 event or any single WHO stage 4 event.

By WHO stage 3 events are much more common and as such the groups are more similar. They also occur much earlier
