# Supplementary material for: Multiple HIV-1 infections with evidence of recombination in heterosexual partnerships in a low risk Rural Clinical Cohort in Uganda
Source: Virology. 2011 Mar 1;411(1):113–31. doi: 10.1016/j.virol.2010.12.025 (PMC3041926; doi:10.1016/j.virol.2010.12.025)
Supplement: Supp. Table 3b — Rate of disease outcomes in incident multiple and singly infected individuals any single WHO stage 3 event or any single WHO stage 4 event. [file mmc6.doc]

**Table 3b. Rate of disease outcomes in incident multiple and singly infected individuals**

|  | Singly infected  (N = 22) | | Multiple infected  (N = 4) | | Crude Hazard Ratio  (95% CI) | P value |
| --- | --- | --- | --- | --- | --- | --- |
|  | n (%) | Rate per  100y (95% CI) | n (%) | Rate per  100y (95% CI) |
| Mortality | 6 (27%) | 3.4 (1.5, 7.6) | 2 (50%) | 5.0 (1.3, 20.1) | 1.92 (0.35, 10.49) | 0.472 |
| CD4≤250^a^ | 11 (52%) | 10.4 (5.8, 18.8) | 4 (100%) | 18.1 (6.8, 48.2) | 1.66 (0.52, 5.29) | 0.413 |
| Received ART^b^ | 8 (38%) | 5.2 (2.6, 10.5) | 2 (50%) | 6.3 (1.6, 25.2) | 1.22 (0.25, 5.96) | 0.809 |
| WHO disease events^c, d^ | 11 (50%) | 19.8 (9.9- 39.7) | 4 (100%) | 25.0 (9.4-66.6) | 1.92 (0.47-7.78) | 0.36 |

^a^ One singly infected individuals excluded from analysis as CD4 count≤250 at time of first HIV test

^b^ One singly infected individual excluded from analysis as started ART at time of first HIV test

^c^ One singly infected individuals excluded from analysis as experienced WHO stage events at time of first HIV test

^d^ Any single WHO stage 3 event or any single WHO stage 4 event.
